# The distinct responsiveness of cytokeratin 19-positive hepatocellular carcinoma to regorafenib

**DOI:** 10.1038/s41419-021-04320-4

**Published:** 2021-11-16

**Authors:** Jianyong Zhuo, Di Lu, Zuyuan Lin, Xinyu Yang, Modan Yang, Jianguo Wang, Yaoye Tao, Xue Wen, Huihui Li, Zhengxing Lian, Beini Cen, Siyi Dong, Xuyong Wei, Haiyang Xie, Shusen Zheng, Youqing Shen, Xiao Xu

**Affiliations:** 1 Department of Hepatobiliary and Pancreatic Surgery, Center for Integrated Oncology and Precision Medicine, the Affiliated Hangzhou First People’s Hospital, Zhejiang University School of Medicine, Hangzhou, China; 2 Institute of Organ Transplantation, Zhejiang University School of Medicine, Hangzhou, China; 3 Department of Pathology, the First Affiliated Hospital, Zhejiang University School of Medicine, Hangzhou, China; 4 National Center for Healthcare Quality Management in Liver Transplant, Hangzhou, China; 5 Department of Hepatobiliary and Pancreatic Surgery, the First Affiliated Hospital, Zhejiang University School of Medicine, Hangzhou, China; 6 National Health Commission Key Laboratory of Combined Multi-organ Transplantation, Hangzhou, China; 7 Department of Hepatobiliary and Pancreatic Surgery, Shulan (Hangzhou) Hospital, Hangzhou, China; 8 Center for Bionanoengineering and Key Laboratory of Biomass Chemical Engineering of Ministry of Education, College of Chemical and Biological Engineering, Zhejiang University, Hangzhou, China

**Keywords:** Targeted therapies, Liver cancer

## Abstract

Cytokeratin 19-positive (CK19+) hepatocellular carcinoma (HCC) is an aggressive subtype characterized by early recurrence and chemotherapy tolerance. However, there is no specific therapeutic option for CK19+ HCC. The correlation between tumor recurrence and expression status of CK19 were studied in 206 patients undergoing liver transplantation for HCC. CK19−/+ HCC cells were isolated to screen effective antitumor drugs. The therapeutic effects of regorafenib were evaluated in patient-derived xenograft (PDX) models from 10 HCC patients. The mechanism of regorafenib on CK19+ HCC was investigated. CK19 positiveness indicated aggressiveness of tumor and higher recurrence risk of HCC after liver transplantation. The isolated CK19+ HCC cells had more aggressive behaviors than CK19− cells. Regorafenib preferentially increased the growth inhibition and apoptosis of CK19+ cells in vitro, whereas sorafenib, apatinib, and 5-fluorouracil did not. In PDX models from CK19−/+ HCC patients, the tumor control rate of regorafenib achieved 80% for CK19+ HCCs, whereas 0% for CK19− HCCs. RNA-sequencing revealed that CK19+ cells had elevated expression of mitochondrial ribosomal proteins, which are essential for mitochondrial function. Further experiments confirmed that regorafenib attenuated the mitochondrial respiratory capacity in CK19+ cells. However, the mitochondrial respiration in CK19− cells were faint and hardly repressed by regorafenib. The mitochondrial respiration was regulated by the phosphorylation of signal transducer and activator of transcription 3 (STAT3), which was inhibited by regorafenib in CK19+ cells. Hence, CK19 could be a potential marker of the therapeutic benefit of regorafenib, which facilitates the individualized therapy for HCC. STAT3/mitochondria axis determines the distinct response of CK19+ cells to regorafenib treatment.

## Introduction

Cytokeratin 19 (CK19) is a marker for early hepatoblasts, hepatic progenitor cells, and cholangiocytes [1, 2]. As summarized in our previous review, there are 10–30% of hepatocellular carcinoma (HCC) presents CK19-positive expression [3]. CK19-positive (CK19+) HCC is related to poor tumor differentiation, tumor recurrence and metastasis, as well as poor prognosis [4–9]. In addition, the CK19-related gene signature has strongly overlapped with previously described more aggressive HCC subclass, such as “Hoshida_S2” [10], “Chiang_Proliferation” [11], “iCluster1 subtype” [12], and “Shimada_MS1” [13]. Using microarrays and microRNA profiling in a Caucasian cohort of 242 consecutive HCC samples, Govaere et al. [14] reported the distinct molecular profile of CK19+ HCCs, which was different from other HCC types. Taking these into consideration, CK19+ HCC should be diagnosed and treated as a unique subtype. Although various studies have described the prognostic relevance of CK19 in HCCs, there are no effective drugs for this subtype. In addition, CK19+ HCC have been validated to be resistant to chemotherapy, such as doxorubicin and 5-fluorouracil [14, 15]. Therefore, it is necessary to identify specific therapeutic options for CK19+ HCC.

Patient-derived xenograft (PDX) models are generated through directly implanting freshly patient-derived tumor tissues into immunodeficient mice. PDX models retain the principal histological characteristics, recapitulate the genetic, transcriptomic, and proteomic characteristics, making PDXs closely correlating with molecular phenotypes of their donor patients [16, 17]. Therefore, in preclinical research and clinical translation studies, PDX models have been widely used to identify effective options for personalized treatments [18, 19].

In this study, we constructed a “CK19 promoter-driven expressed green fluorescence protein” report system in accordance with a previous report [15] and isolated CK19-negative (CK19−) and CK19+ cells by fluorescence activated cell sorting (FACS). Furthermore, we assessed the responses of CK19+ and CK19− cells to sorafenib (SOR), regorafenib (REG), apatinib (APA), and 5-fluorouracil (5FU). We also validated the efficacy of regorafenib in HCC PDX models from five patients with CK19− tumors and five patients with CK19+ tumors. Our findings suggested that regorafenib should be considered as a potential option for individualized therapy in patients with CK19+ HCC.

## Materials and methods

### Patients and samples

For clinical survival analysis, 206 tumor samples were collected from patients with HCC who had underwent liver transplantation at the First Affiliated Hospital, Zhejiang University School of Medicine between January 2015 and December 2018. The follow-up was ended on August 31, 2020. The procedures of this study were in accordance with the Declaration of Helsinki. For HCC PDXs, fresh HCC tumor samples were obtained from patients with HCC who underwent surgical resection. All patients were fully informed and provided written informed consent. All study protocols were approved by the Ethics Committee of the First Affiliated Hospital, Zhejiang University School of Medicine.

### Cell culture

Human HCC cell lines Huh7 and PLC/PRL/5 were obtained from the Type Culture Collection of the Chinese Academy of Sciences (Shanghai, China). The cell lines were cultured in Dulbecco’s modified Eagle’s medium supplemented with 10% fetal bovine serum (Gibco, USA) and 1% penicillin/streptomycin at 37 °C in 5% CO_2_.

### Construction of a CK19− green fluorescent protein (GFP) reporter

The CK19 promoter sequence was generated and cloned into plasmid vector that expressed GFP under the control of the CK19 promoter as described previously [15].

### Cell sorting

The plasmid vector was transfected into human HCC cells according to the manufacturer’s instructions. To sort CK19− and CK19+ cells, stably transfected cells were sorted according to GFP expression by FACS (Beckman Coulter, Miami, FL, USA).

### CCK-8 cell viability assays

Cells were seeded into 96-well plates (3000 cells/well) with 100 μL medium and incubated overnight. Then, cells were treated with the negative control or the indicated drugs for 72 h. At the time of analysis, 10 μL CCK-8 reagent (HY-K0301; MedChem Express, Monmouth Junction, NJ, USA) was added to each well. After 2 h of incubation, the OD values were determined using a microplate reader (BioTek, Winooski, VT, USA) at a wavelength of 450 nm.

### Colony formation assay

Cells were seeded into 6-well plates (1000 cells/well) with 2 mL medium and cultured for 14 days. Then, we counted the numbers of colonies in each well.

### Apoptosis assays

Cells were treated with the negative control or with the indicated drugs for 48 h. At the time of analysis, cells were trypsinized and incubated with binding buffer containing APC and 7-AAD (640930; BioLegend, San Diego, CA, USA). After 15 min of incubation at room temperature in the dark, the apoptosis rate was measured using flow cytometer (BD Biosciences, San Jose, CA, USA).

### Agents and confirmation of working concentration

The drugs used in the current study included sorafenib (SOR, S7397; Selleck, Houston, TX, USA), regorafenib (REG, S1178; Selleck), apatinib (APA, a kindly gift from Hengrui Medicine Co., Ltd.), and 5-fluorouracil (5FU, F6627, Sigma; St. Louis, MO, USA). Drugs were dissolved in dimethyl sulfoxide (DMSO) and diluted with the medium to the desired concentration.

To determine the half-inhibitory concentration (IC50), Huh7 and PLC/PRF/5 cells were cultured with SOR, REG, APA, and 5FU, respectively, for 48 h at a series of concentrations. The working concentration of each drug was defined as a half of IC50 in Huh7 or PLC/PRF/5 cells for subsequent experiments.

### Animals

Five-week-old, male, NOD/SCID/IL-2γ-receptor-null (NSG) mice were used to establish PDXs, and 5-week-old, male, nude mice were used for further evaluation of efficacy. All mice were acclimated with a 12-hour light/dark cycle under specific pathogen-free conditions. All animal protocols were approved by the Animal Experiment Ethical Committee of the First Affiliated Hospital, Zhejiang University School of Medicine. All mice were cared for in accordance with the Guide for the Care and Use of Laboratory Animals of the National Institutes of Health.

### Establishment of PDX models

PDX models were established in NSG mice, as previously described [20]. Briefly, surgically resected HCC tissues sized approximately 2 mm were subcutaneously implanted into the flanks of NSG mice. When a tumor volume reached ~1000 mm^3^, the mouse was sacrificed and PDX tumor tissues were extracted and implanted into nude mice for in vivo pharmacological studies.

### In vivo efficacy evaluation

For efficacy evaluation of drugs, mice were randomly divided into the negative control group, REG group (20 mg/kg/day), SOR group (30 mg/kg/day), APA group (100 mg/kg/day), and 5FU group (25 mg/kg/3 day) with five mice per group, when the tumor volume was 50–200 mm^3^. For evaluation of the efficacy of regorafenib, mice from each PDX model were treated with or without REG (20 mg/kg/day; *n* = 3–5 per group) when the tumor volume reached approximately 50–200 mm^3^. The tumor sizes and body weights of the mice were measured twice a week. Tumor volume was calculated as length × width^2^ × 0.5. The relative tumor volume (RTV) of each tumor was calculated as the ratio of the volume on a specified observation day to the volume at the start of therapy. The tumor growth inhibition (TGI) for each PDX model was calculated as follows: TGI = (1 − [mean RTV in the treated group]/[mean RTV in the control group]) × 100%. Three categories of REG responses were defined as follows: (a) response, >80% TGI; (b) stability, 50–80% TGI; and (c) nonresponse, <50% TGI; and tumor control rate is calculated as follows: tumor control rate = (response cases + stability cases)/(response cases + stability cases + nonresponse cases) × 100%.

### Immunohistochemistry and TUNEL assay

Tumor tissue samples were stained using anti-CK19 and anti-Ki67. The immunohistochemistry procedures for CK19 and Ki-67 were performed by the Department of Pathology, the First Affiliated Hospital, Zhejiang University School of Medicine. Expression of CK19 with moderate or strong intensity in >5% of tumor cells was defined as CK19 positivity. The nuclear fraction of Ki67 positivity in tumor cells was quantitatively measured. Cell apoptosis in tumor sections were evaluated by Terminal deoxynucleotidyl transferase-mediated dUTP Nick End-Labeling (TUNEL) using the TUNEL staining kit (C1089; Beyotime, Shanghai, China). Apoptotic nucleuses were visualized with fluorescent microscopy.

### Quantitative real-time PCR

Total RNA was extracted from cells using an RNA-Quick Purification Kit (RN001; ES Science, Shanghai, China), and cDNA was synthesized according to the manufacturer’s instructions. Quantitative real-time polymerase chain reaction (qRT-PCR) was performed using QuantStudio5 (Thermo, Waltham, MA, USA). The primers used in the current study are detailed in Supplementary Table 1.

### Western blotting

Cells were lysed in RIPA buffer containing proteinase inhibitors. The proteins were separated using 10% SDS-PAGE gels and transferred to polyvinylidene difluoride membranes, as previously reported [21]. The membranes were incubated at 4 °C overnight with primary antibodies (anti-CK19 (1:1000; 10712-1-AP; Proteintech, Wuhan, China), anti-Signal transducer and activator of transcription 3 (STAT3) (1:1000; 12640; Cell Signaling Technology, Danvers, MA, USA), anti-phospho-STAT3 (1:1000; 9145; Cell Signaling Technology), and anti-GAPDH (1:5000; EM1101; Huabio, Hangzhou, China)). The bands were visualized using FluorChem M (Bio-Techne, Minneapolis, MN, USA).

### RNA sequencing

Cells were treated with or without regorafenib, and total RNA was extracted. Sequencing libraries were generated using the NEBNext Ultra RNA Library Prep Kit for Illumina (NEB, USA) following the manufacturer’s recommendations. RNA sequencing was performed by Novogene (Beijing, China) using the Illumina HiSeq platform. Differential expression analysis of two groups was performed using the DESeq2 package under R software. Adjusted *P* value of 0.05 and absolute fold change of 0 were set as the threshold for significantly differential expression. Gene Ontology (GO) and KEGG enrichment analysis were implemented by the clusterProfiler R package.

### Mitochondrial morphology

Cells were plated into confocal dishes and treated with regorafenib for 48 h. At the time of analysis, cells were washed once with phosphate-buffered saline (PBS) and stained with 50 nM Mito-Tracker Red CMXRos (M7512; ThermoFisher, Waltham, MA, USA) at 37 °C in a 5% CO_2_ humidified incubator for 30 min, followed by staining with DAPI at room temperature for 10 min. Mitochondrial morphology was determined by confocal microscopy (Leica, Wetzlar, Germany). The diameter (max) and diameter (min) of mitochondria in groups were measured by Image-pro plus, and the length index was calculated as follows: length index = diameter (max)/diameter (min).

### Oxygen consumption rate and extracellular acidification rate assays

The oxygen consumption rate (OCR) and extracellular acidification rate (ECAR) were measured using a Seahorse XFe96 extracellular flux analyzer (Agilent Technologies, Santa Clara, CA, USA) according to the manufacturer’s instructions. Cells were treated with or without regorafenib for 48 h, then 10,000 cells were plated into XF Cell Culture Microplates and incubated overnight. For OCR assay, oligomycin A (2.5 μM), FCCP (0.5 μM), and rotenone/antimycin A (0.5 μM) were injected into each well sequentially at the indicated time points; for ECAR, glucose (10 mM), oligomycin (1 μM), and 2-DG (50 mM) were sequentially injected. The final data were normalized to the number of cells.

### Small interfering RNA transfection

Small interfering RNA (siRNA) were obtained from GuanNan Co., Ltd. (Hangzhou, China). Cells were transfected with STAT3 siRNA or control RNA according to the manufacturer’s instructions, the sequence is shown in Supplementary Table 2.

### Statistical analysis

Statistical analyses were performed using GraphPad Prism (Version 6; GraphPad, La Jolla, CA, USA) and SPSS (Version 23; IBM, Armonk, NY, USA). All statistical analyses were performed using the unpaired two-sided *t* tests. The Kaplan-Meier method and log-rank test were used for survival analysis. All the results represented three or more independent experiments with the data expressed as mean ± SD. The results with *P* values less than 0.05 were considered significant.

## Results

### Clinicopathological and prognostic relevance of CK19 in HCC

**Fig. 1:**
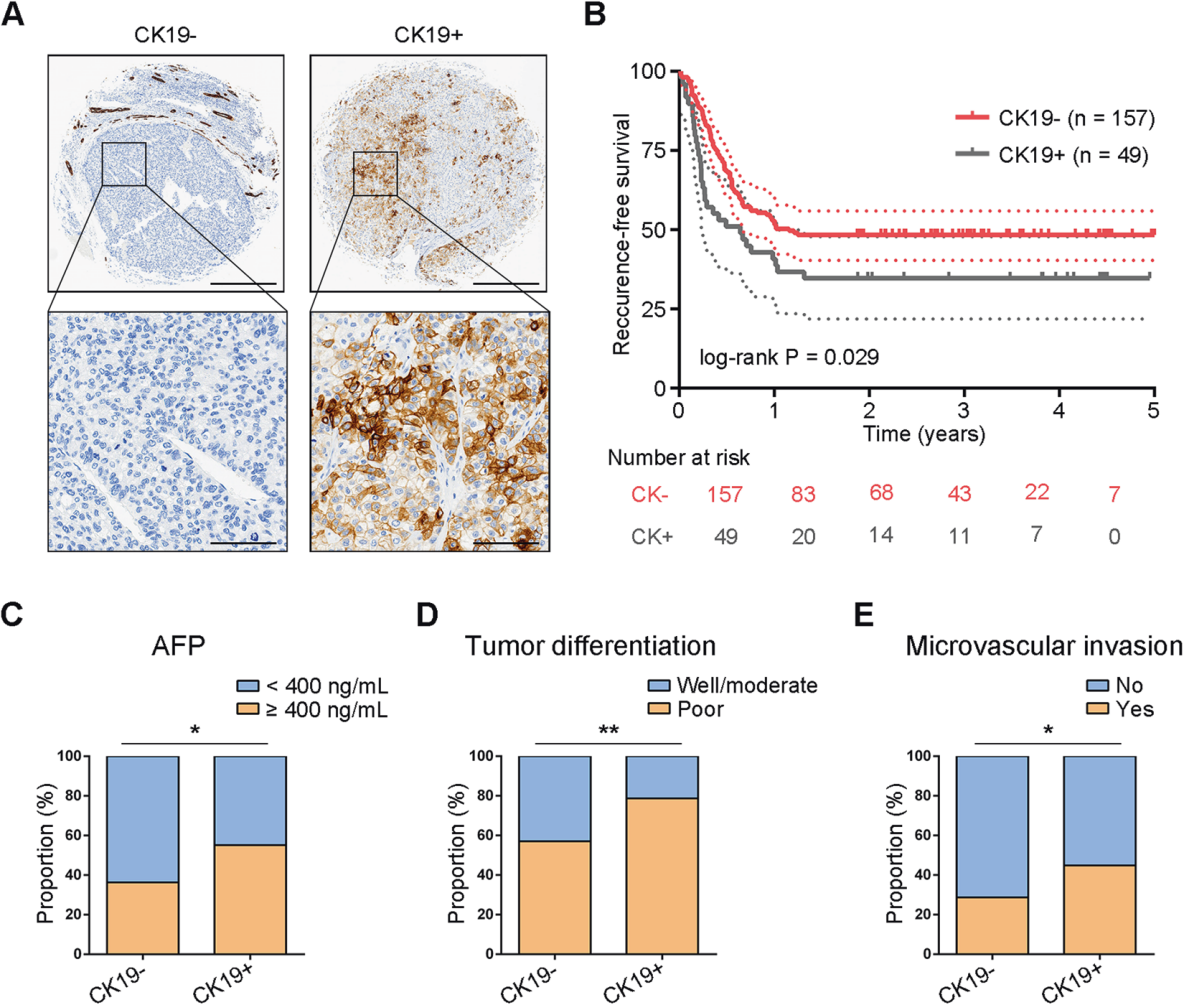
Clinicopathological characteristics of patients with hepatocellular carcinoma based on CK19 expression. **A** Representative images of CK19-negative and CK19-positive expression in HCC tissues, as detected by immunohistochemistry. Scale bars, 500 μm (upper panel), 125 μm (lower panel). **B** Kaplan-Meier survival curves showing recurrence-free survival in patients with CK19− HCC (*n* = 157) and CK19+ HCC (*n* = 49). **C**–**E** Significantly different clinicopathological characteristics between the CK19− and CK19+ groups, Chi-square test, ^*^P < 0.05 and ^**^P < 0.01. HCC, hepatocellular carcinoma; CK19−, CK19-negative; CK19+, CK19-positive.

To assess clinicopathological and prognostic relevance of CK19, a cohort of 206 patients with HCC who had undergone liver transplantation were retrospectively analyzed. Immunohistochemistry data showed that CK19 presented in 23.8% (49/206) patients (Fig. 1A). Survival data showed that liver recipients with CK19+ HCC had significantly lower recurrence-free survival (RFS) than recipients with CK19− HCC (*P* = 0.029, Fig. 1B). The 1- and 3-year RFS rates were, respectively, 40.8% and 34.7% for CK19+ patients, and 52.9%, and 48.4% for CK19− patients, respectively. CK19 presence was related to elevated preoperative alpha fetoprotein (AFP) levels (*P* = 0.019), poor tumor differentiation (*P* = 0.007), and microvascular invasion (*P* = 0.034, Fig. 1C–E and Table 1). Consistent with previous studies [15, 22], these data suggest that liver recipients with CK19+ HCC have a higher likelihood of tumor recurrence after liver transplantation.

**Table 1:** Clinicopathological characteristics of patients with CK19− and CK19+ hepatocellular carcinoma.

	CK19- (*n* = 157)	CK19+ (*n* = 49)	*P* value
Sex			0.893
Male	142 (90.4%)	44 (89.8%)	
Female	15 (9.6%)	5 (10.2%)	
Age (years)			0.797
≤50	80 (51.0%)	26 (53.1%)	
>50	77 (49.0%)	23 (46.9%)	
HbsAg			0.314
Positive	150 (95.5%)	45 (91.8%)	
Negative	7 (4.5%)	4 (8.2%)	
Liver cirrhosis			0.910
Yes	150 (95.5%)	47 (95.9%)	
No	7 (4.5%)	2 (4.1%)	
AFP (ng/mL)			0.019
<400	100 (63.7%)	22 (44.9%)	
≥400	57 (36.3%)	27 (55.1%)	
Tumor differentiation^a^			0.007
Well/moderate	65 (43.0%)	10 (21.3%)	
Poor	86 (57.0%)	37 (78.7%)	
Tumor size (cm)^b^			0.465
≤5	90 (60.0%)	22 (53.7%)	
>5	60 (40.0%)	19 (46.3%)	
Tumor number^c^			0.892
≤3	106 (68.4%)	33 (67.3%)	
>3	49 (31.6%)	16 (32.7%)	
Microvascular invasion			0.034
Yes	45 (28.7%)	22 (44.9%)	
No	112 (71.3%)	27 (55.1%)	

The data were analyzed by Chi-square tests. CK19−, CK19-negative; CK19+, CK19-positive; HbsAg, Hepatitis B surface antigen; AFP, serum alpha fetoprotein.

^a^There were eight cases of missing data.

^b^There were 15 cases of missing data.

^c^There were two cases of missing data.

### CK19+ HCC cells increased malignant properties

**Fig. 2:**
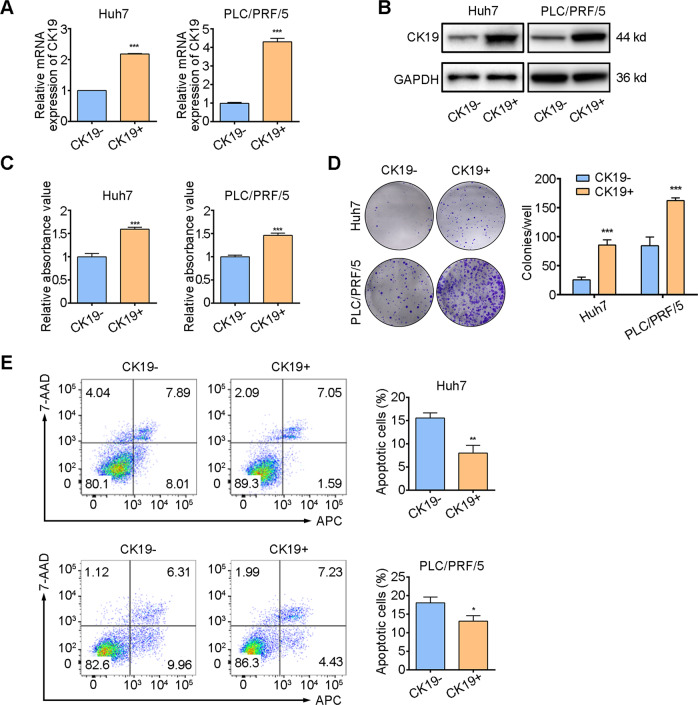
Phenotype validation after construction of a CK19 promoter report system. **A** qPCR and **B** immunoblotting detection in CK19− and CK19+ HCC of Huh7 and PLC/PRF/5 cells. **C** CCK-8 assay, **D** colonial formation, and **E** cell apoptosis assay in CK19− and CK19+ of Huh7 and PLC/PRF/5 cells. The data are shown as mean ± SD. ^*^P < 0.05, ^**^P < 0.01, and ^***^P < 0.001. CCK-8, cell counting kit-8; HCC, hepatocellular carcinoma; CK19−, CK19-negative; CK19+, CK19-positive.

To obtain CK19+ and CK19− HCC cells, we generated a CK19-EGFP reporter vector under the control of CK19 promoter as previously described [15]. Huh7 and PLC/PRF/5 human HCC cell lines were transfected with the CK19-EGFP reporter vector, and the CK19+ and CK19− cells were isolated by FACS according to the EGFP fluorescence. The qPCR and western blotting results showed that CK19+ cells had higher mRNA and protein expression of CK19 than CK19− cells (Fig. 2A, B). Compared with CK19− cells, CK19+ cells exhibited higher proliferative ability and higher capacity to form colonies (Fig. 2C, D). In addition, CK19+ cells had less tumor cell apoptosis than CK19− cells (Fig. 2E). Taken together, these results suggest that CK19+ HCC cells have higher malignant potential than CK19− cells.

### CK19+ HCC cells were more sensitive to regorafenib than CK19− cells

**Fig. 3:**
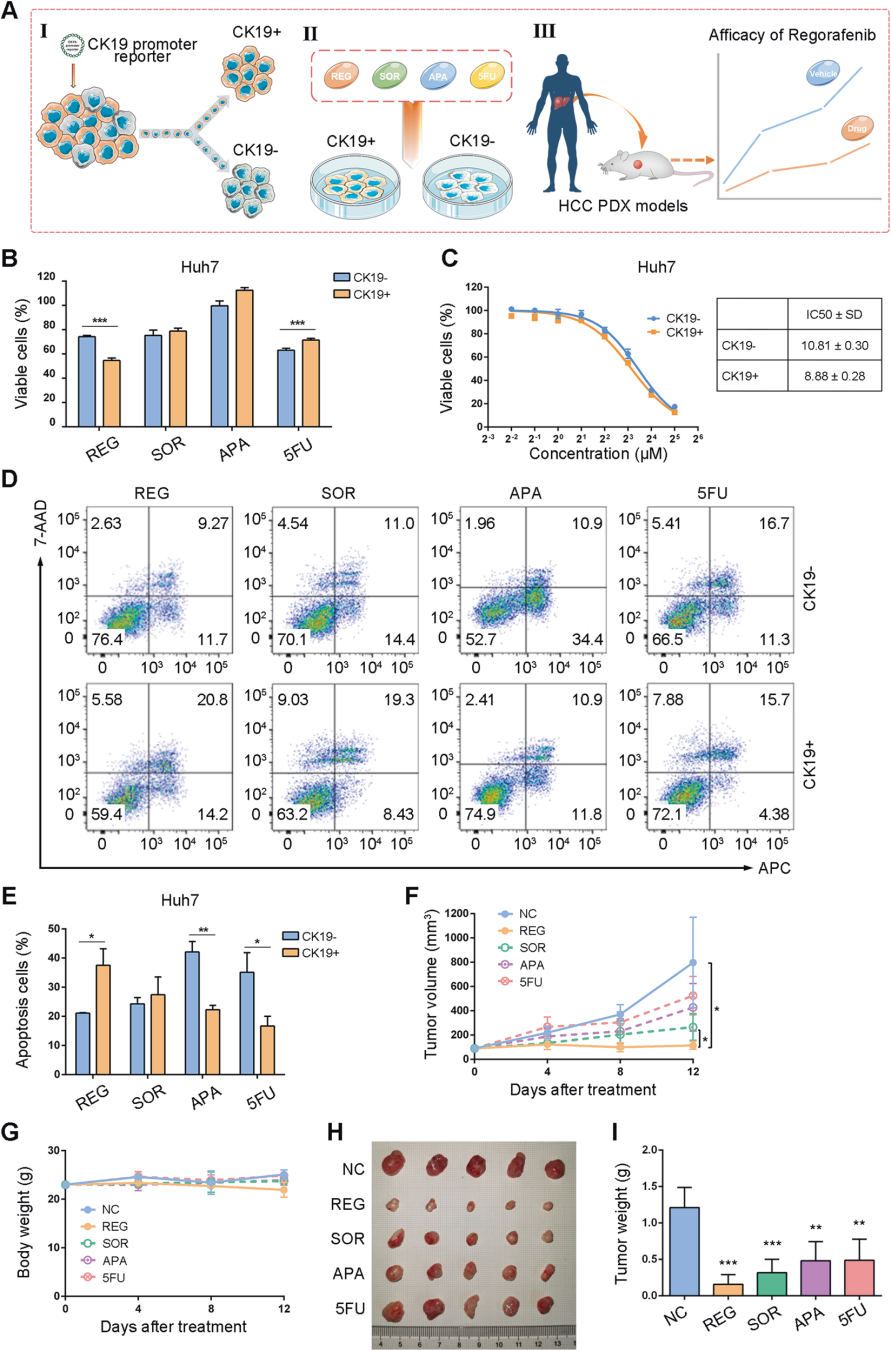
Responses of CK19− and CK19+ cells to different anti-cancer drugs. **A** Cell sorting workflow after construction of a CK19-promoter report system in HCC cells (I). After sorting, CK19− and CK19+ HCC cells were treated with REG, SOR, APA, and 5Fu to screen for drug sensitivity in CK19+ cells (II). HCC patient-derived xenografts (PDXs) were used to validate the efficacy of regorafenib (III). **B** CK19− and CK19+ of Huh7 cells were treated with REG, SOR, APA, and 5Fu (*n* = 4). The effects were evaluated and compared with corresponding untreated cells using CCK-8 assays. The results are shown as the percent viable cells. **C** IC50 values of REG in CK19− and CK19+ Huh7 cells. **D**, **E** CK19− and CK19+ of Huh7 cells were treated with REG, SOR, APA, and 5Fu (*n* = 3), and the proportion of apoptotic cells was measured using flow cytometric analysis. CK19+ hepatocellular carcinoma PDXs were used to evaluate the efficacy of REG, SOR, APA, and 5Fu. Mice were randomly divided into negative control (NC), REG (20 mg/kg), SOR (30 mg/kg), APA (100 mg/kg), and 5Fu (25 mg/kg) groups (*n* = 5). **F** Tumor growth curves in each group. **G** Body weights of mice in each group. **H** Photographic images of tumors from PDX mice in response to different treatments. **I** Tumor weights in each group, and the tumor weights of indicated drug treatment groups were compared to that in negative group (NC). Data are shown as mean ± SD. ^*^P < 0.05, ^**^P < 0.01, and ^***^P < 0.001. CK19−, CK19-negative; CK19+, CK19-positive; CCK-8, cell counting kit-8; IC50, half-inhibitory concentration; REG, regorafenib; SOR, sorafenib; APA, apatinib; 5Fu, 5-fluorouracil.

Next, we investigated the effects of targeted agents and chemotherapeutic agents on CK19−/+ HCC cells. As shown in the schematic flow (Fig. 3A), we treated CK19+ and CK19− cells with or without SOR, REG, APA, and 5FU, respectively, at the indicated concentration (Supplementary Fig. S1). The cytotoxic assays showed that REG suppressed the proliferation of CK19+ cells with a higher efficacy compared with CK19− cells (Fig. 3B and Supplementary Fig. S2A). The IC50 values of REG in CK19+ cells were lower than these in CK19− cells (Fig. 3C and Supplementary Fig. S2B). Whereas, CK19+ cells showed less growth inhibition than CK19− cells when treated with SOR, APA, and 5FU (Fig. 3B and Supplementary Fig. S2A). In addition, the apoptosis assays demonstrated that REG treatment resulted in a marked increase in apoptosis in CK19+ cells compared with that in CK19− cells, whereas apoptosis rate were lower in CK19+ cells than in CK19− cells after treatment with SOR, APA, and 5FU (Fig. 3D, E and Supplementary Fig. S2C, D). We also evaluated the efficacy of SOR, REG, APA, and 5FU in PDX-bearing mice from a patient with CK19+ HCC. Interestingly, REG had the greatest effects on tumor inhibition (Fig. 3F–I). Collectively, these data demonstrate that CK19+ HCC cells have higher sensitivity to REG treatment than CK19− cells.

### CK19+ HCC PDX models showed better responses to regorafenib

**Fig. 4:**
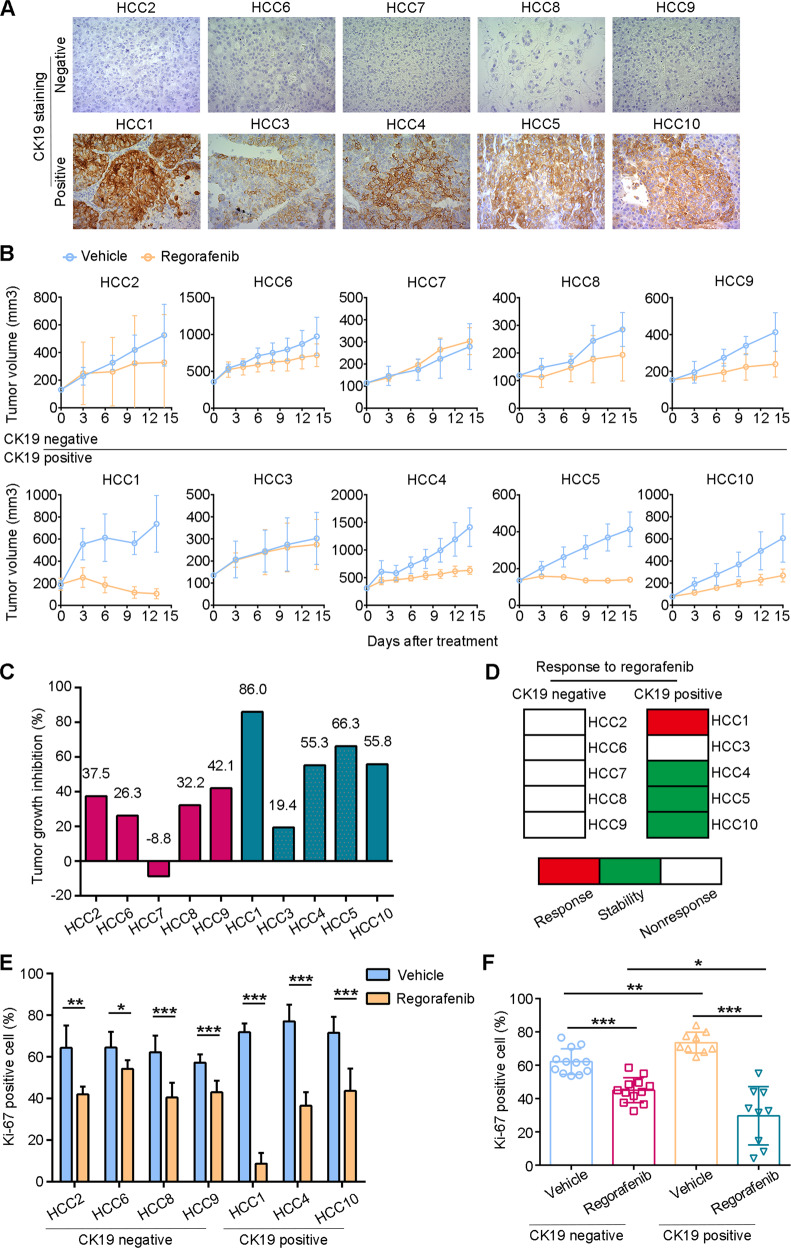
Various efficacy of regorafenib in HCC patient-derived xenografts. **A** Images of CK19 expression detected using immunohistochemistry in tumors from 10 patients with HCC. Magnification, 400×. **B** Tumor growth curves in 10 PDX models. **C** Quantitative TGI in CK19− PDXs (pink bar) and CK19+ PDXs (blue bar). TGI was calculated as TGI = 1 – (Δ*T*/Δ*C*) (%), where Δ*T* indicates the fold change in the regorafenib treatment group, and Δ*C* indicates the fold change in the negative control group. **D** Responses of 10 PDXs to regorafenib treatment. We defined >80% TGI as regorafenib response (red block), 50–80% TGI as stability (green block), and <50% TGI as nonresponse (white block). **E** The Ki67-positive proportion of tumors in regorafenib group was compared with that in vehicle group in each PDX model case (*n* = 3). **F** The integrated comparison of Ki67-positive proportion from CK19− PDX tumors treated with vehicle (*n* = 12), CK19− PDX tumors treated with regorafenib (*n* = 12), CK19+ PDX tumors treated with vehicle (*n* = 9), and CK19+ PDX tumors treated with regorafenib (*n* = 9). Data are shown as mean ± SD. ^*^P < 0.05, ^**^P < 0.01, and ^***^P < 0.001. CK19−, CK19-negative; CK19+, CK19-positive; PDX, patient-derived xenograft; TGI, tumor growth inhibition.

To further validate the effects of regorafenib on HCC with different CK19 phenotypes, we established PDX models from HCC tumor tissues of ten patients (five patients with CK19− HCC (CK19− group) and five patients with CK19+ HCC (CK19+ group)) (Fig. 4A). The clinicopathological characteristics of patients in CK19− group and CK19+ group were summarized in Supplementary Table 3. For each patient, the corresponding PDX mice with appropriate tumor volumes were randomly allocated into the vehicle and the regorafenib groups (3–5 mice per group). The results indicated that the efficacy of regorafenib varied in these ten PDX models (Fig. 4B). The TGI indexes were, respectively, 85.98%, 66.27%, 55.80%, 55.25%, and 19.4% in the five CK19+ HCC PDX models, and 42.05%, 37.47%, 32.24%, 26.28%, and −8.84% in the five CK19− HCC PDX models (Fig. 4C). The average TGI in the CK19+ group tended to be lower than that in the CK19− group (56.54% versus 25.84%, *P* = 0.0614, Supplementary Fig. S3A). We further defined TGI greater than 80% as response, between 50% and 80% as stability, and less than 50% as nonresponse, in accordance with previously published methods [23]. In the CK19+ group, one patient achieved tumor response, three patients showed stability, and only one patient showed tumor nonresponse; whereas in the CK19− group, all five patients showed tumor nonresponse to regorafenib. The tumor control rates were 80% (4 of 5) in the CK19+ group and 0% (0 of 5) in the CK19− PDX group (Fig. 4D). Additionally, we also measured the proliferative and apoptotic levels in tumors of both the vehicle group and the regorafenib group. The data demonstrated that the proportion of Ki67-positive nuclei in CK19+ tumors was significantly lower than that in CK19− tumors after regorafenib treatment (Fig. 4E, F); and the TUNEL assay revealed that CK19+ tumors had significantly more cell apoptosis after regorafenib treatment in comparison to CK19− tumors (Supplementary Fig. S3B–D). Taken together, these results indicate that the inhibitory effects of regorafenib on CK19+ HCC are greater than those in CK19− HCC.

### Mitochondrial ribosomal proteins were involved in the response of CK19+ HCC cells to regorafenib

**Fig. 5:**
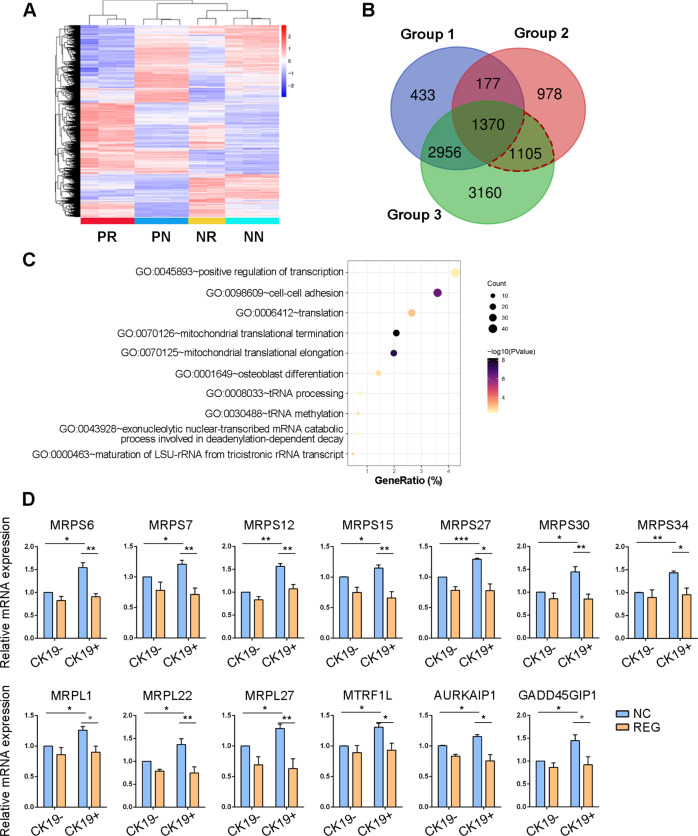
Mitochondria was involved in mediating the sensitivity of CK19 + cells to regorafenib. **A** Heatmap of DEGs in the four groups. PR, CK19-positive cells treated with regorafenib; PN, CK19-positive cells as a negative control; NR, CK19-negative cells treated with regorafenib; NN, CK19-negative cells as a negative control. **B** Venn diagram of three groups of DEGs. Group 1, DEGs between NR and NN; group 2, upregulated genes from DEGs between PN and NN; group 3, regulated genes from DEGs between PR and PN. **C** Top 10 enriched biological processes by GO analysis. **D** Relative mRNA expression of mitochondrial ribosomal proteins (MRPs) in CK19− and CK19+ cells treated with control and regorafenib. The data are shown as mean ± SD. ^*^P < 0.05, ^**^P < 0.01, and ^***^P < 0.001. NC, negative control; REG, regorafenib; CK19−, CK19-negative; CK19+, CK19-positive; DEGs, differentially expressed genes.

Because of the specific inhibitory effects of regorafenib on CK19+ cells, we further investigated the underlying mechanisms. We performed RNA sequencing for four groups, including CK19− cells treated with DMSO as negative control (NN), CK19− cells treated with regorafenib (NR), CK19+ cells treated with DMSO as negative control (PN), and CK19+ cells treated with regorafenib (PR). We obtained differentially expressed genes (DEGs) through a binary comparison between two groups (Fig. 5A) and extracted a set of genes using the following route: (1) obtain DEGs between NR and NN groups (group 1); (2) extract upregulated genes from DEGs between PN and NN groups (group 2); (3) extract significantly regulated genes from DEGs between PR and PN groups (group 3); and (4) extract genes from the intersection of group 2 and group 3 and then exclude genes presented in group 1. Finally, we obtained a set of 1105 genes, which indicated these genes were specifically regulated by regorafenib in CK19+ cells (Fig. 5B). In this gene set, GO analysis showed that mitochondrial translational termination and mitochondrial translational elongation were the two most prominent biological processes, in which many genes encoding mitochondrial ribosomal proteins (MRPs) are enriched (Fig. 5C and Supplementary Table 4). Furthermore, we validated the RNA sequencing results by using qPCR. The qPCR results showed that CK19+ cells had higher mRNA expression of MRPs than CK19− cells; and compared with that of the corresponding negative control, regorafenib treatment significantly reduced the mRNA expression of MRPs in CK19+ cells, whereas only a few MRPs were decreased in CK19− cells (Fig. 5D). The data indicate that MRPs play crucial roles in the distinctly inhibitory effect of regorafenib on CK19+ cells.

### Regorafenib downregulated mitochondrial respiration in CK19+ HCC cells

**Fig. 6:**
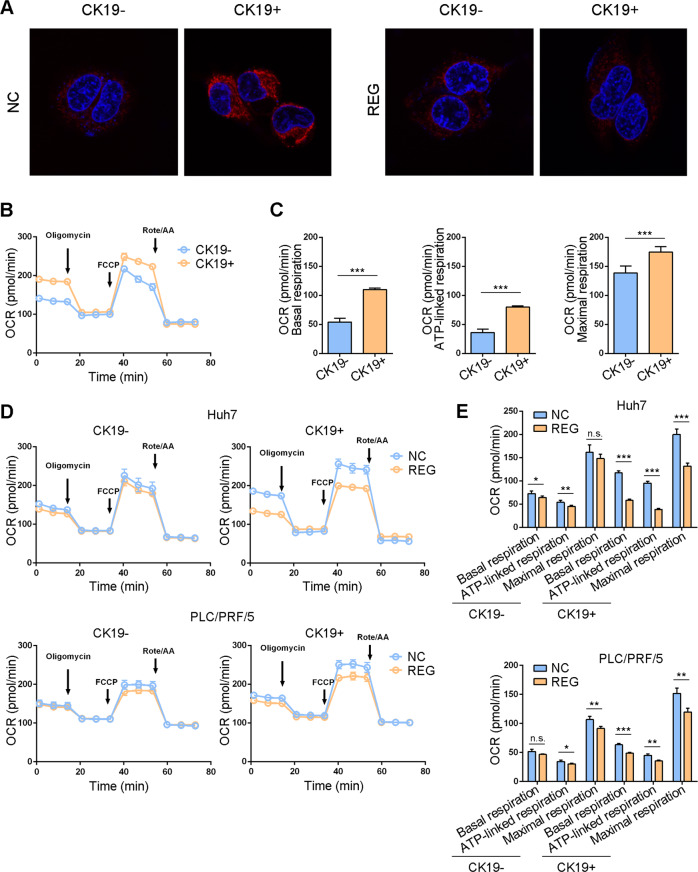
Mitochondrial respiration was inhibited by regorafenib in CK19 + cells. **A** Mitochondrial morphologies in CK19− and CK19+ cells treated with or without regorafenib were observed by confocal microscopy. Magnification, 1800×. **B** OCRs of CK19− and CK19+ Huh7 cells were measured using a Seahorse XFp extracellular flux analyzer and were normalized to the number of cells. **C** The quantitation of key OCR parameters, including basal respiration, ATP-linked respiration and maximal respiration, in CK19− and CK19+ Huh7 cells. **D** OCRs of CK19− cells and CK19+ cells treated with or without regorafenib were measured and normalized to the number of cells. **E** The quantitation of key OCR parameters in CK19− and CK19+ cells. Data are presented as mean ± SD, n.s., not significant, ^*^P < 0.05, ^**^P < 0.01, and ^***^P < 0.001. NC, negative control; REG, regorafenib; CK19−, CK19-negative; CK19+, CK19-positive; OCR, oxygen consumption rate.

Mitochondrial ribosomes are essential in the regulation of mitochondrial respiration [24, 25]. Therefore, we investigated whether regorafenib could regulate the mitochondrial respiration in CK19+ HCC cells. Using confocal microscopy, we observed that mitochondria had increased fusion and elongation in CK19+ Huh7 cells than that in CK19− Huh7 cells. After treating with regorafenib, CK19+ Huh7 cells exhibited punctate mitochondria and disorganized cristae (Fig. 6A and Supplementary Fig. S4). Moreover, we measured the mitochondrial respiration by mitochondrial stress-testing with a Seahorse analyzer. The data showed that CK19+ Huh7 cells had a higher OCR that CK19− Huh7 cells (Fig. 6B, C). Regorafenib treatment dramatically reduced the OCR in CK19+ cells, but not in CK19− cells (Fig. 6D, E); and Seahorse-based ECAR assay showed that CK19+ cells had higher glycolysis than CK19− cells. CK19+ cells treated with regorafenib significantly elevated the glycolysis level compared to the control, however, CK19− cells had limited increase in the glycolysis level after regorafenib treatment (Supplementary Fig. S5A–C). We also drew an energy map according to the basal OCR and EACR to visualize the energy change in the cells (Supplementary Fig. S5D). Taken together, these data indicate that CK19+ cells have increased mitochondrial respiratory capacity compared with CK19− cells. Regorafenib treatment downregulates the mitochondrial function in CK19+ cells.

### Dephosphorylated STAT3 mediated the inhibition of mitochondrial respiration by regorafenib

**Fig. 7:**
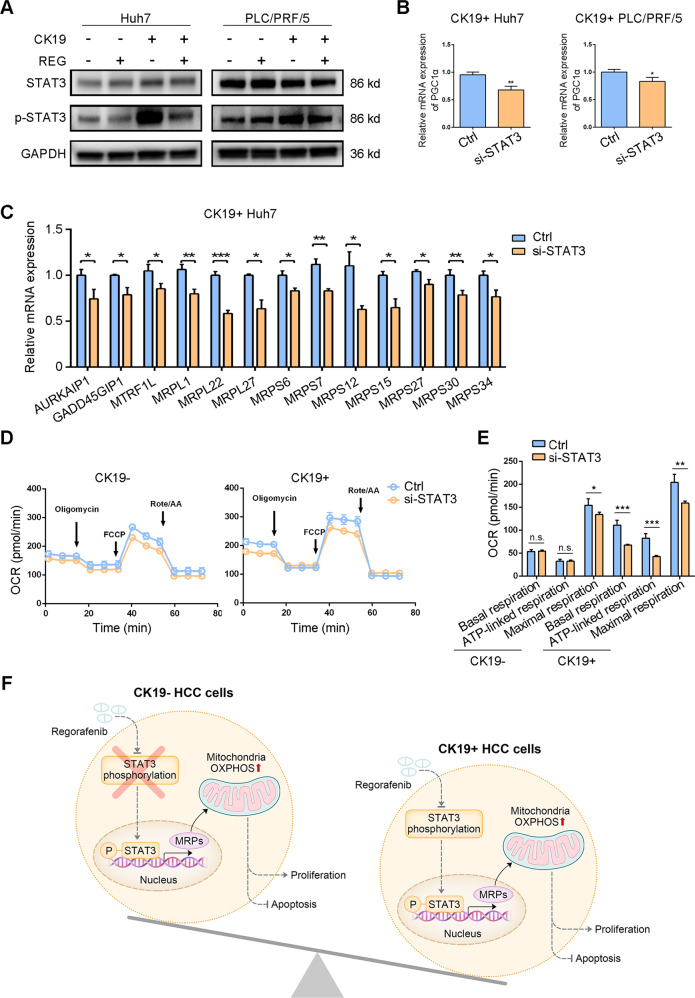
Phosphorylated STAT3 mediate the mitochondrial function. **A** Immunoblot analysis of protein extracts from CK19− and CK19+ cells treated with or without REG, GAPDH was used as a loading control. **B** The mRNA expression of PGC-1α after STAT3 knockdown in CK19+ Huh7 and PLC/PRF/5 cells. **C** The mRNA expression of mitochondrial ribosomal proteins changed after knockdown of STAT3 in CK19+ Huh7 cells. **D** OCRs of CK19− cells and CK19+ cells transfected with ctrl and si-STAT3 were measured and normalized to the number of Huh7 cells. **E** The quantitation of key OCR parameters in CK19− and CK19+ Huh7 cells transfected with ctrl and si-STAT3. **F** A theoretic model indicated the distinct effect of regorafenib on CK19+ hepatocellular carcinoma via STAT3-dependent modulation of mitochondrial function. Data are presented as mean ± SD, n.s., not significant, ^*^P < 0.05, ^**^P < 0.01, and ^***^P < 0.001. Ctrl, control; REG, regorafenib; CK19−, CK19-negative; CK19+, CK19-positive; STAT3, signal transducer and activator of transcription 3; MRPs, mitochondrial ribosomal proteins; OXPHOS, oxidative phosphorylation.

STAT3 is reported to promote the mitochondrial respiration in cancer stem cells (CSC) [26, 27]. Moreover, Tai et al. [28] reported that phosphorylation of STAT3 is an important target of regorafenib in HCC. Thus, we hypothesized that regorafenib inhibited mitochondrial respiration of CK19+ cells via STAT3 signaling. We put these 1105 specially regulated genes from Fig. 5B into online KnockTF analysis, and the result indicated that STAT3 was one of the significant transcription factors regulated by regorafenib in CK19+ cells (Supplementary Table 5). The western blotting results showed that compared with CK19− cells, CK19+ HCC cells had increased phosphorylation of STAT3 at Tyr705, and STAT3 Tyr705 phosphorylation was decreased by regorafenib treatment in CK19+ cells (Fig. 7A). Knockdown of STAT3 by siRNA transfection inhibited the mRNA level of peroxisome proliferator-activated receptor γ coactivator 1-α (*PGC-1α*), a nodal regulator of mitochondrial biogenesis [29] (Fig. 7B and Supplementary Fig. S6). More importantly, STAT3 knockdown significantly reduced the mRNA level of MRPs in CK19+ cells (Fig. 7C). STAT3 knockdown also decreased the OCR level in CK19+ Huh7 cells, but not in CK19− Huh7 cells (Fig. 7D, E). Thus, these findings suggest that regorafenib treatment exerts inhibitory effects on mitochondrial function through inactivation of STAT3 Tyr705 phosphorylation in CK19+ cells (Fig. 7F).

## Discussion

CK19 is a cytoskeletal intermediate filament and is present at normal biliary/hepatic progenitor cells [30, 31]. As summarized in our previous review, CK19 occurs in 10–30% of HCCs, and CK19+ HCCs are associated with worse overall survival and early tumor recurrence after hepatectomy and liver transplantation [3]. In the present study, our data were consistent with this description, 49 of 206 (23.8%) patients in our cohort exhibited CK19+ HCC. Patients with CK19+ tumors had lower RFS than patients with CK19− tumors after undergoing liver transplantation. CK19 plays a crucial role in tumor invasion and chemotherapeutic resistance in HCC [3, 14]. However, the optimal therapeutic strategy for CK19+ HCC remains unclear. Hence, we constructed CK19− and CK19+ cell lines and PDX models to identify specific inhibitors of CK19+ HCC and clarify the underlying mechanisms.

In this study, we found that the proliferation of CK19− cells was inhibited to a greater extent by SOR, APA, and 5FU than that of CK19+ cells. Similarly, Kawai et al. [15] showed that CK19+ cells were significantly more resistant to 5FU than CK19− cells. Govaere et al. [14] also reported that knockdown of CK19 rendered HCC cells more sensitive to doxorubicin, fluorouracil, and sorafenib. Thus, CK19+ cells may be more resistant to chemotherapy and sorafenib. Unexpectedly, in our study, we found that regorafenib inhibited the proliferation of CK19+ cells and promoted apoptosis in CK19+ cells to a greater extent. We also explored the therapeutic effect of regorafenib in HCC PDX models. We found that regorafenib exhibited the greatest efficacy in a CK19+ HCC PDX model when compared with vehicle and other treatments including SOR, APA, and 5FU. Furthermore, in 10 HCC PDXs from patients with different CK19 expression statuses of tumors, we observed that CK19+ HCC PDX models had higher responsiveness to regorafenib than their CK19− counterparts. Thus, data from cell lines and PDXs suggested that CK19+ cells were more sensitive to regorafenib treatment.

Regorafenib, an oral multi-kinase inhibitor, blocks various targets, including those involved in angiogenesis (VEGFR1–3 and TIE2), stromal functions (PDGFR-β and FGFR), and oncogenesis (KIT, RET, and RAF) [32]. Despite similarities of the chemical structures between sorafenib and regorafenib, the effects and molecular mechanism of regorafenib are expected to be different from those of sorafenib [33]. For example, Tai et al. reported that regorafenib was a more potent inhibitor of STAT3 than sorafenib [28]. Regorafenib has been approved as a second-line therapy for patients who show disease progression despite the use of sorafenib [34]. An international multicenter retrospective study also demonstrated the safety of regorafenib in sorafenib-tolerant patients with HCC recurrence after liver transplantation [35]. More interestingly, Zhu et al. [36]. reported that some treatment-naive patient-derived HCC xenograft models showed better responses to regorafenib than sorafenib. How to optimize the therapeutic sequence of sorafenib and regorafenib deserves more investigation. Our data support an idea that regorafenib may be an effective first-line treatment for patients with CK19+ HCC. However, more extensive preclinical/clinical studies are needed.

Tumor metabolism plays an essential role in the growth and survival of cancer cells. The bulk of tumor cells are thought to acquire energy predominantly from aerobic glycolysis or the Warburg effect [37]. However, recent studies have shown that mitochondrial oxidative phosphorylation plays a crucial role in the bioenergetics of CSCs [38–40]. Increasing evidence indicates that CSCs use mitochondrial respiration preferentially to maintain stem-like properties [39, 41, 42]. For example, in HCC, Wei et al. [43] reported that a subpopulation of Nanog-positive cells exhibiting CSC-like features showed enhanced mitochondrial respiration. CK19 has been identified as another important CSC marker for HCC [14, 15, 44]. Therefore, we inferred that CK19+ cells may also rely on mitochondrial respiration. In this study, bioinformatics analysis suggested that the special efficacy of regorafenib in CK19+ HCC was mainly attributed to mitochondrial translation. We also showed that CK19+ cells had higher mRNA levels of MRP genes and OCR. When compared with CK19− cells, CK19+ cells showed greater downregulation of MRP gene expression and OCR after treated with regorafenib. However, CK19+ cells have both high-OXPHOS and glycolytic lever. Unexpectedly, CK19+ cells also elevated the glycolytic level after regorafenib treatment. We infer that the metabolic balance in CK19+ cells is perturbed by regorafenib despite the glycolytic change. Of course, the metabolic shift in CK19+ cells is very interesting and deserves further investigations.

The transcription factor STAT3 upregulates mitochondrial transcripts and enhances oxidative metabolism [26]. Canonical STAT3 activation by phosphorylation of Tyr705 site, which is involved in nuclear translocation, promotes stem cell-like characteristics, survival, and proliferation [45]. Tai et al. [28] reported that regorafenib was a potent inhibitor of STAT3, functioning by blocking STAT3-related signaling and enhancing HCC inhibition by reducing phospho-STAT3 signaling. Consistent with the above findings, we demonstrated that CK19+ cells exhibited increased STAT3 Tyr705 phosphorylation than CK19− cells, and this increased STAT3 phosphorylation in CK19+ cells were dramatically induced by regorafenib treatment. Moreover, we demonstrated that downregulation of STAT3 by siRNA inhibited the OCR and MRPs in CK19+ cells. Taken together, these data indicated that regorafenib inhibited the CK19+ HCC by disrupting mitochondrial function via STAT3 signaling. But it is not excluded that regorafenib inhibited CK19+ HCC through other potential mechanisms, as it is a multiple kinase inhibitor. For example, Govaere’s study demonstrated that CK19+ HCC elevated the gene expression of platelet-derived growth factor receptor alpha (PDGFRα) compared to CK19− HCC, and PDGFRα could improve invasion and metastasis of CK19+ HCC [14, 46]. As PDGFRα is one of multiple targets of regorafenib, their findings imply PDGFRα pathway may contribute to the specific effect of regorafenib on CK19+ HCC as well.

In conclusion, our data demonstrated a crucial role of CK19 phenotype in predicting poor prognosis and preserving the malignant properties of HCC. This definite subtype of HCC showed remarkable responses to regorafenib. Therefore, our study offers strong evidence that regorafenib should be used as an individualized therapy for CK19+ HCC and that this therapeutic regimen merits further investigation in clinical trials.

## Supplementary information

Supplemental material.
