# Supplementary material for: The distinct responsiveness of cytokeratin 19-positive hepatocellular carcinoma to regorafenib
Source: Cell Death Dis. 2021 Nov 16;12(12):1084. doi: 10.1038/s41419-021-04320-4 (PMC8595883; doi:10.1038/s41419-021-04320-4)

**Supplementary Materials**

**Supplementary Table 1.** The primer sequences used in the current study. Primer sequence of genes were obtained from PrimerBank (https://pga.mgh.harvard.edu/primerbank).

| **Genes** | **Primer sequence (5' -> 3')** | |
| --- | --- | --- |
| MRPL1 | Forward primer | AGGTGCATGGGTAGAGCCTT |
|  | Reverse primer | GGGCACTCGGATGTTTACAGA |
| MRPL14 | Forward primer | ACTGGGAGTCTGAGTGCGATT |
|  | Reverse primer | AGGAGCCCGATGGTATGGG |
| MRPL17 | Forward primer | CCGCGTATTTCGCCGTATG |
|  | Reverse primer | CATGGTGCCTCGATGCGTT |
| MRPL19 | Forward primer | GGAATGTTATCGAAGGACAAGGT |
|  | Reverse primer | TCCAGCCGTTTCTCTAATTTGAC |
| MRPL21 | Forward primer | ATCTTGTTCGAGTAGAAGCCACA |
|  | Reverse primer | GGGTCGTGACGATTCTTTTCTT |
| MRPL22 | Forward primer | CGAGGAATGTCTATTGACCAGGC |
|  | Reverse primer | CGGATGCGTTTCAGGCACT |
| MRPL27 | Forward primer | GAAGGTCACTATGTTCATGCTGG |
|  | Reverse primer | AGTGTAGCGGACTATCCCCTC |
| MRPL30 | Forward primer | TTGCGCTTAGTAGTTCAATGGC |
|  | Reverse primer | TGGTGAATTTGTGACGAATCCAA |
| MRPL37 | Forward primer | TCCCCTGGATAGGGTGTACG |
|  | Reverse primer | GAGCGGTAGAACCTTGGGT |
| MRPL49 | Forward primer | GCTACCATGTTCCGGGCTAC |
|  | Reverse primer | CAGGCGCTCCACAAACTGATA |
| MRPS6 | Forward primer | GGCCAGAGACTGCTGCTAC |
|  | Reverse primer | GCGGTGGGTGCATAAAAATCC |
| MRPS7 | Forward primer | AGGCAACCATCGAACGCAA |
|  | Reverse primer | CCAGCCCAATCATAGGCTCAC |
| MRPS12 | Forward primer | ACTCAGCCAATCGCAAGTG |
|  | Reverse primer | CGCCCTCCACAAGGACAAT |
| MRPS15 | Forward primer | GGACGCTGAGTTTGATTCGGA |
|  | Reverse primer | GCCCCACTGGTTGAAAGGA |
| MRPS27 | Forward primer | GGAGAGGAACTTTGGTGCATC |
|  | Reverse primer | GCATAGCCATACAACTGGGAAC |
| MRPS30 | Forward primer | AGAGCGAGGTCATATCTTTGCC |
|  | Reverse primer | ACCACGCACCCAGTAAAAATG |
| MRPS34 | Forward primer | CTCCGGGCCATGATTATCGC |
|  | Reverse primer | CCATGCGTATCCTCTGCACAT |
| MTRF1L | Forward primer | CCGCGTCCATACTAGCACC |
|  | Reverse primer | GCTCGCTTAGTGTCAATTCTCAA |
| AURKAIP1 | Forward primer | CAGACTGGATACCGGGACC |
|  | Reverse primer | CTTCAGCACGTTTTTGCACTG |
| GADD45GIP1 | Forward primer | GCACGCAGCCTACTAGGTG |
|  | Reverse primer | CGAACTGCTTAGCCGCGTA |
| GFM2 | Forward primer | GCAGGCAAAACTACCACCAC |
|  | Reverse primer | TCTTGGGCCATGAAATCTGTC |
| KRT19 | Forward primer | AACGGCGAGCTAGAGGTGA |
|  | Reverse primer | GGATGGTCGTGTAGTAGTGGC |
| STAT3 | Forward primer | CAGCAGCTTGACACACGGTA |
|  | Reverse primer | AAACACCAAAGTGGCATGTGA |
| PGC1A | Forward primer | TCTGAGTCTGTATGGAGTGACAT |
|  | Reverse primer | CCAAGTCGTTCACATCTAGTTCA |
| GAPDH | Forward primer | GGAGCGAGATCCCTCCAAAAT |
|  | Reverse primer | GGCTGTTGTCATACTTCTCATGG |

**Supplementary Table 2.** The sequence of STAT3 siRNA used in the present study.

| **siRNA** | **siRNA sequence (5' -> 3')** |
| --- | --- |
| STAT3 si-1 sense | CCCGUCAACAAAUUAAGAA |
| STAT3 si-2 sense | GCGUCCAGUUCACUACUAA |
| STAT3 si-3 sense | GCCUCUCUGCAGAAUUCAA |
| si N-CTL sense | UUCUCCGAACGUGUCACGU |

**Supplementary Table 3.** The clinicopathological characteristics of patients in CK19- and CK19+ group.

| **PDX ID** | **CK19** | **Gender** | **Age (years)** | **AFP prior to surgery** | **HbsAg** | **Liver cirrhosis** |
| --- | --- | --- | --- | --- | --- | --- |
| HCC2 | － | M | 59 | 134.0 | ＋ | ＋ |
| HCC6 | － | M | 58 | 1.4 | ＋ | ＋ |
| HCC7 | － | M | 48 | 47244.1 | ＋ | － |
| HCC8 | － | M | 53 | 5.8 | ＋ | － |
| HCC9 | － | M | 70 | 10.9 | ＋ | － |
| ­HCC1 | ＋ | M | 49 | 674.1 | ＋ | ＋ |
| HCC3 | ＋ | F | 55 | 292.3 | ＋ | － |
| HCC4 | ＋ | M | 32 | 80000.0 | ＋ | ＋ |
| HCC5 | ＋ | M | 57 | 1860.7 | ＋ | ＋ |
| HCC10 | ＋ | M | 51 | 2.3 | ＋ | － |

**(continue)**

| **PDX ID** | **CK19** | **Source** | **Treatment prior to surgery** | **Tumor size (cm)** | **Tumor differentiation** | **Microvascular invasion** |
| --- | --- | --- | --- | --- | --- | --- |
| HCC2 | － | Primary | None | 2.2 | Poor | No |
| HCC6 | － | Primary | None | 1.7 | Moderate | No |
| HCC7 | － | Primary | None | 5.2 | Poor | No |
| HCC8 | － | Primary | None | 3.8 | Poor | No |
| HCC9 | － | Primary | None | 6.6 | Poor | Yes |
| ­HCC1 | ＋ | Primary | None | 2.0 | Moderate | Yes |
| HCC3 | ＋ | Primary | None | 1.6 | Poor | No |
| HCC4 | ＋ | Primary | None | 8.0 | Poor | Yes |
| HCC5 | ＋ | Primary | None | 5.5 | Poor | Yes |
| HCC10 | ＋ | Primary | None | 4.0 | Poor | Yes |

Note: HCC, hepatocellular carcinoma; CK19-, CK19 negative; CK19+, CK19 positive; AFP, serum alpha fetoprotein; TACE, transarterial chemoembolization.

**Supplementary Table 4.** Gene Oncology enrichment analysis of specially regulated genes in CK19-positive cells in term of biological process.

| Term | Count | PValue | Fold Enrichment |
| --- | --- | --- | --- |
| GO:0070126~mitochondrial translational termination | 22 | 6.39E-09 | 4.599173 |
| GO:0070125~mitochondrial translational elongation | 21 | 2.88E-08 | 4.441768 |
| GO:0098609~cell-cell adhesion | 38 | 3.80E-07 | 2.520983 |
| GO:0006412~translation | 28 | 8.81E-04 | 1.989725 |
| GO:0000463~maturation of LSU-rRNA from tricistronic rRNA transcript (SSU-rRNA, 5.8S rRNA, LSU-rRNA) | 5 | 9.53E-04 | 9.988104 |
| GO:0001649~osteoblast differentiation | 15 | 0.001812 | 2.593065 |
| GO:0030488~tRNA methylation | 7 | 0.002555 | 4.840389 |
| GO:0045893~positive regulation of transcription, DNA-templated | 45 | 0.003031 | 1.570944 |
| GO:0008033~tRNA processing | 8 | 0.003803 | 3.887262 |
| GO:0043928~exonucleolytic nuclear-transcribed mRNA catabolic process involved in deadenylation-dependent decay | 7 | 0.004573 | 4.339659 |
| GO:0036498~IRE1-mediated unfolded protein response | 10 | 0.004988 | 3.047218 |
| GO:0051289~protein homotetramerization | 10 | 0.005587 | 2.996431 |
| GO:0070208~protein heterotrimerization | 5 | 0.006054 | 6.420924 |
| GO:0030308~negative regulation of cell growth | 15 | 0.00725 | 2.22875 |
| GO:0006364~rRNA processing | 22 | 0.008217 | 1.848266 |
| GO:0007049~cell cycle | 22 | 0.009558 | 1.822714 |
| GO:0071158~positive regulation of cell cycle arrest | 6 | 0.010985 | 4.314861 |
| GO:0042273~ribosomal large subunit biogenesis | 6 | 0.010985 | 4.314861 |
| GO:0008380~RNA splicing | 18 | 0.011011 | 1.949485 |
| GO:0071902~positive regulation of protein serine/threonine kinase activity | 7 | 0.011779 | 3.595717 |
| GO:0000398~mRNA splicing, via spliceosome | 22 | 0.012183 | 1.781662 |
| GO:0006672~ceramide metabolic process | 5 | 0.012603 | 5.28782 |
| GO:0030033~microvillus assembly | 5 | 0.012603 | 5.28782 |
| GO:0000245~spliceosomal complex assembly | 6 | 0.012993 | 4.148905 |
| GO:0042059~negative regulation of epidermal growth factor receptor signaling pathway | 7 | 0.013488 | 3.495836 |
| GO:0006884~cell volume homeostasis | 4 | 0.015288 | 7.191435 |
| GO:0032502~developmental process | 5 | 0.015505 | 4.994052 |
| GO:0006887~exocytosis | 11 | 0.016458 | 2.382704 |
| GO:0045351~type I interferon biosynthetic process | 3 | 0.017165 | 13.48394 |
| GO:0042271~susceptibility to natural killer cell mediated cytotoxicity | 3 | 0.017165 | 13.48394 |
| GO:0038026~reelin-mediated signaling pathway | 3 | 0.017165 | 13.48394 |
| GO:2001237~negative regulation of extrinsic apoptotic signaling pathway | 7 | 0.01741 | 3.311845 |
| GO:0001568~blood vessel development | 7 | 0.01741 | 3.311845 |
| GO:0010033~response to organic substance | 6 | 0.017719 | 3.852554 |
| GO:0043523~regulation of neuron apoptotic process | 5 | 0.018795 | 4.731207 |
| GO:0006892~post-Golgi vesicle-mediated transport | 5 | 0.018795 | 4.731207 |
| GO:0048662~negative regulation of smooth muscle cell proliferation | 6 | 0.020458 | 3.719708 |
| GO:0015031~protein transport | 33 | 0.020908 | 1.502009 |
| GO:0008283~cell proliferation | 31 | 0.021296 | 1.522776 |
| GO:0006397~mRNA processing | 18 | 0.021751 | 1.807903 |
| GO:0098656~anion transmembrane transport | 6 | 0.023459 | 3.595717 |
| GO:0030212~hyaluronan metabolic process | 4 | 0.025796 | 5.992862 |
| GO:0042407~cristae formation | 4 | 0.025796 | 5.992862 |
| GO:0005980~glycogen catabolic process | 5 | 0.026586 | 4.280616 |
| GO:0070431~nucleotide-binding oligomerization domain containing 2 signaling pathway | 3 | 0.02756 | 10.78715 |
| GO:0046329~negative regulation of JNK cascade | 5 | 0.031105 | 4.086042 |
| GO:0048268~clathrin coat assembly | 4 | 0.032179 | 5.531873 |
| GO:0089711~L-glutamate transmembrane transport | 4 | 0.032179 | 5.531873 |
| GO:0007265~Ras protein signal transduction | 9 | 0.039684 | 2.311533 |
| GO:0021517~ventral spinal cord development | 3 | 0.039834 | 8.989293 |
| GO:0043320~natural killer cell degranulation | 3 | 0.039834 | 8.989293 |
| GO:0010887~negative regulation of cholesterol storage | 3 | 0.039834 | 8.989293 |
| GO:0001501~skeletal system development | 14 | 0.041418 | 1.837228 |
| GO:0009615~response to virus | 12 | 0.042011 | 1.9613 |
| GO:0031100~organ regeneration | 7 | 0.044599 | 2.677662 |
| GO:0007015~actin filament organization | 9 | 0.045737 | 2.247323 |
| GO:0030214~hyaluronan catabolic process | 4 | 0.047156 | 4.79429 |
| GO:0006122~mitochondrial electron transport, ubiquinol to cytochrome c | 4 | 0.047156 | 4.79429 |
| GO:0000470~maturation of LSU-rRNA | 4 | 0.047156 | 4.79429 |
| GO:0031167~rRNA methylation | 4 | 0.047156 | 4.79429 |
| GO:0007213~G-protein coupled acetylcholine receptor signaling pathway | 4 | 0.047156 | 4.79429 |
| GO:0034198~cellular response to amino acid starvation | 5 | 0.047215 | 3.595717 |
| GO:0032543~mitochondrial translation | 6 | 0.047333 | 2.996431 |
| GO:0048013~ephrin receptor signaling pathway | 10 | 0.048959 | 2.090533 |
| GO:0001822~kidney development | 10 | 0.048959 | 2.090533 |

**Supplementary Table 5.** The potential transcription factors specifically regulated by regorafenib in CK19+ cells. Regulated genes were analyzed in KnockTF (http://www.licpathway.net/KnockTF/index.html).

| No. | TF | the number of inter genes | p value | FDR |
| --- | --- | --- | --- | --- |
| 1 | FLI1 | 500 | 9.73E-27 | 2.99E-24 |
| 2 | FOXA1 | 419 | 1.49E-14 | 2.29E-12 |
| 3 | TFAP2C | 337 | 1.07E-13 | 1.09E-11 |
| **4** | **STAT3** | **657** | **3.49E-12** | **2.68E-10** |
| 5 | HNRNPLL | 426 | 1.66E-11 | 1.02E-09 |
| 6 | MYCN | 261 | 7.64E-10 | 3.91E-08 |
| 7 | YBX1 | 274 | 1.73E-08 | 7.59E-07 |
| 8 | PRDM14 | 177 | 5.89E-08 | 2.26E-06 |
| 9 | ELK3 | 365 | 3.55E-06 | 1.21E-04 |
| 10 | RBM39 | 481 | 5.59E-06 | 1.72E-04 |
| 11 | ATF2 | 140 | 1.15E-05 | 3.21E-04 |
| 12 | TCF7L1 | 120 | 1.31E-05 | 0.000335 |
| 13 | TARDBP | 657 | 2.74E-05 | 6.47E-04 |
| 14 | XBP1 | 231 | 3.86E-05 | 7.96E-04 |
| 15 | HNF1B | 151 | 3.89E-05 | 7.96E-04 |
| 16 | SOX2 | 414 | 6.29E-05 | 1.21E-03 |
| 17 | SSB | 426 | 1.13E-04 | 2.04E-03 |
| 18 | U2AF2 | 587 | 1.22E-04 | 2.08E-03 |
| 19 | PRPF4 | 256 | 1.73E-04 | 2.80E-03 |
| 20 | MYT1L | 44 | 1.87E-04 | 2.87E-03 |
| 21 | LIN28B | 92 | 0.000228 | 0.00333 |
| 22 | ATM | 234 | 0.000285 | 0.00398 |
| 23 | ZEB2 | 132 | 0.000342 | 0.00456 |
| 24 | SALL4 | 240 | 0.000607 | 0.00776 |
| 25 | GATA6 | 46 | 0.000647 | 0.00795 |
| 26 | PTBP1 | 602 | 0.00138 | 0.0163 |
| 27 | ELF5 | 12 | 0.00195 | 0.0218 |
| 28 | NFATC3 | 583 | 0.00199 | 0.0218 |
| 29 | HES1 | 87 | 0.00219 | 0.0232 |
| 30 | FOXO1 | 183 | 0.0025 | 0.0256 |
| 31 | ESRRG | 68 | 0.00288 | 0.0285 |
| 32 | ARID1A | 122 | 0.00355 | 0.0341 |

**Supplementary Figure Legends**

**Supplementary Figure 1.**

IC50 values of antitumor drugs in HCC cell lines. (A) IC50 of Huh7 for SOR, REG, APA, and 5FU. (A) IC50 of PLC/PRF/5 for SOR, REG, APA, and 5FU. IC50, half-inhibitory concentration; SOR, sorafenib; REG, regorafenib; APA, apatinib; 5FU, 5-fluorouracil. Data were presented as IC50 ± SD.

**Supplementary Figure 2.**

Responses of CK19- and CK19+ of PLC/PRF/5 cells to different anti-cancer drugs. (A) CK19- and CK19+ of PLC/PRF/5 cells were treated with regorafenib (REG), sorafenib (SOR), apatinib (APA) and 5-fluorouracil (5Fu; n = 4). The effects were evaluated and compared with corresponding untreated cells using CCK-8 assays. The results are shown as the percent viable cells. (B) IC50 values of REG in CK19- and CK19+ PLC/PRF/5 cells. (C-D) CK19- and CK19+ of PLC/PRF/5 cells were treated with REG, SOR, APA, and 5Fu (n = 3), and the proportion of apoptotic cells proportion was measured using flow cytometric analysis. Data are shown as means ± SD. *p < 0.05, **p < 0.01, and ***p < 0.001. CK19-, CK19-negative; CK19+, CK19-positive; IC50, half-inhibitory concentration.

**Supplementary Figure 3.**

The pro-apoptosis effect of regorafenib on PDX models. (A) The comparison of TGI in CK19- PDX group (n = 5) and CK19+ PDX group (n = 5), student’s t test, P = 0.0614. (B) Representative images of TUNEL assay in CK19- PDX (HCC2) and CK19+ PDX (HCC1). Scale bar, 20 μm. (C) The quantification of TUNEL assay in each PDX model case treated with vehicle or regorafenib (n = 3). (C) The integrated comparison of TUNEL-positive proportion from CK19- PDX tumors treated with vehicle (n = 12), CK19- PDX tumors treated with regorafenib (n = 12), CK19+ PDX tumors treated with vehicle (n = 9), and CK19+ PDX tumors treated with regorafenib (n = 9). Data are shown as means ± SD, student’s t test, **p < 0.01, and ***p < 0.001; n.s., not significant. TGI, tumor growth inhibition; CK19-, CK19-negative; CK19+, CK19-positive; PDX, patient-derived xenograft; TUNEL, terminal deoxynucleotidyl transferase mediated dUTP nick end labeling.

**Supplementary Figure 4.**

Relative quantification of mitochondrial length in groups. The diameter (max) and diameter (min) of mitochondria in different cells (n=10/group) were measured using Image-Pro Plus, and the length index was calculated as following, length index = diameter (max) / diameter (min). Data were shown as mean ± SD, student’s t test, *p < 0.05, n.s., not significant. NC, negative control; REG, regorafenib.

**Supplementary Figure 5.**

The glycolytic level in CK19- and CK19+ cells. (A) ECARs of CK19- cells and CK19+ cells treated with or without regorafenib were measured and normalized to the number of cells. (B) The quantitation of glycolysis and (C) glycolytic capacity in CK19- and CK19+ cells. Data are presented as the mean ± SD, student’s t test, *p < 0.05, **p < 0.01, and ***p < 0.001, n.s., not significant. (D) The energy map was plotted according to the basal OCR and EACR. Data represent mean ± SD. CK19-, CK19-negative; CK19+, CK19-positive NC, negative control; REG, regorafenib; OCR, oxygen consumption rate; EACR, extracellular acidification rate.

**Supplementary Figure 6.**

The knockdown efficiency of STAT3 siRNAs was tested. (A) Immunoblot analysis of protein extracts from Huh7 and (B) PLC/PRF/5 cells treated with scrambled sequence and three STAT3 siRNA. GAPDH was used as a loading control.

**Supplementary Figure 1.**

**
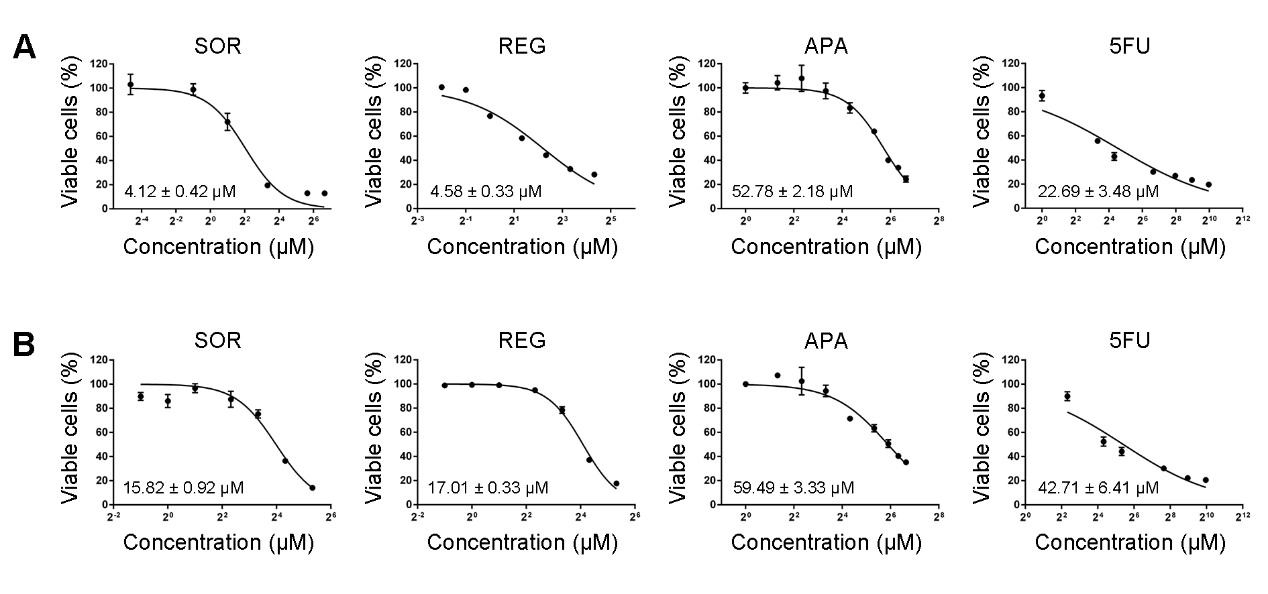
**

**Supplementary Figure 2.**


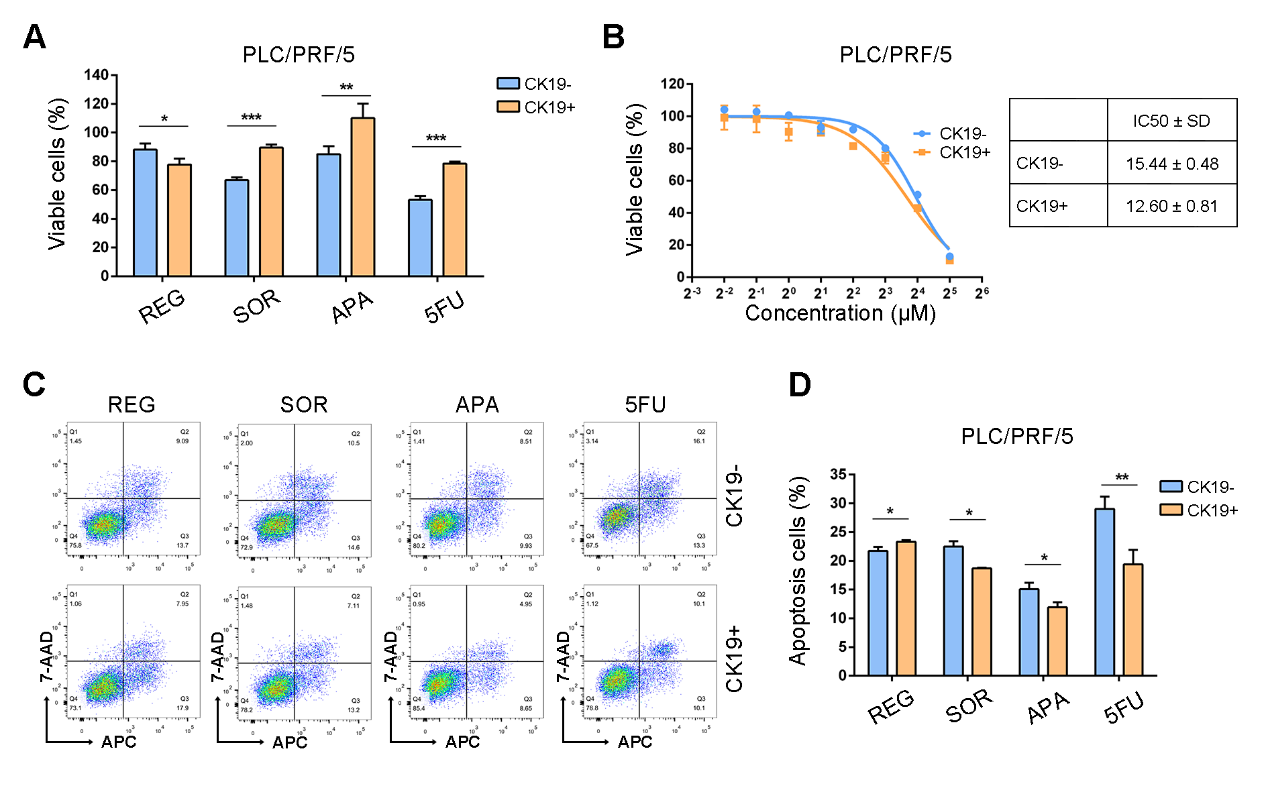


**Supplementary Figure 3.**


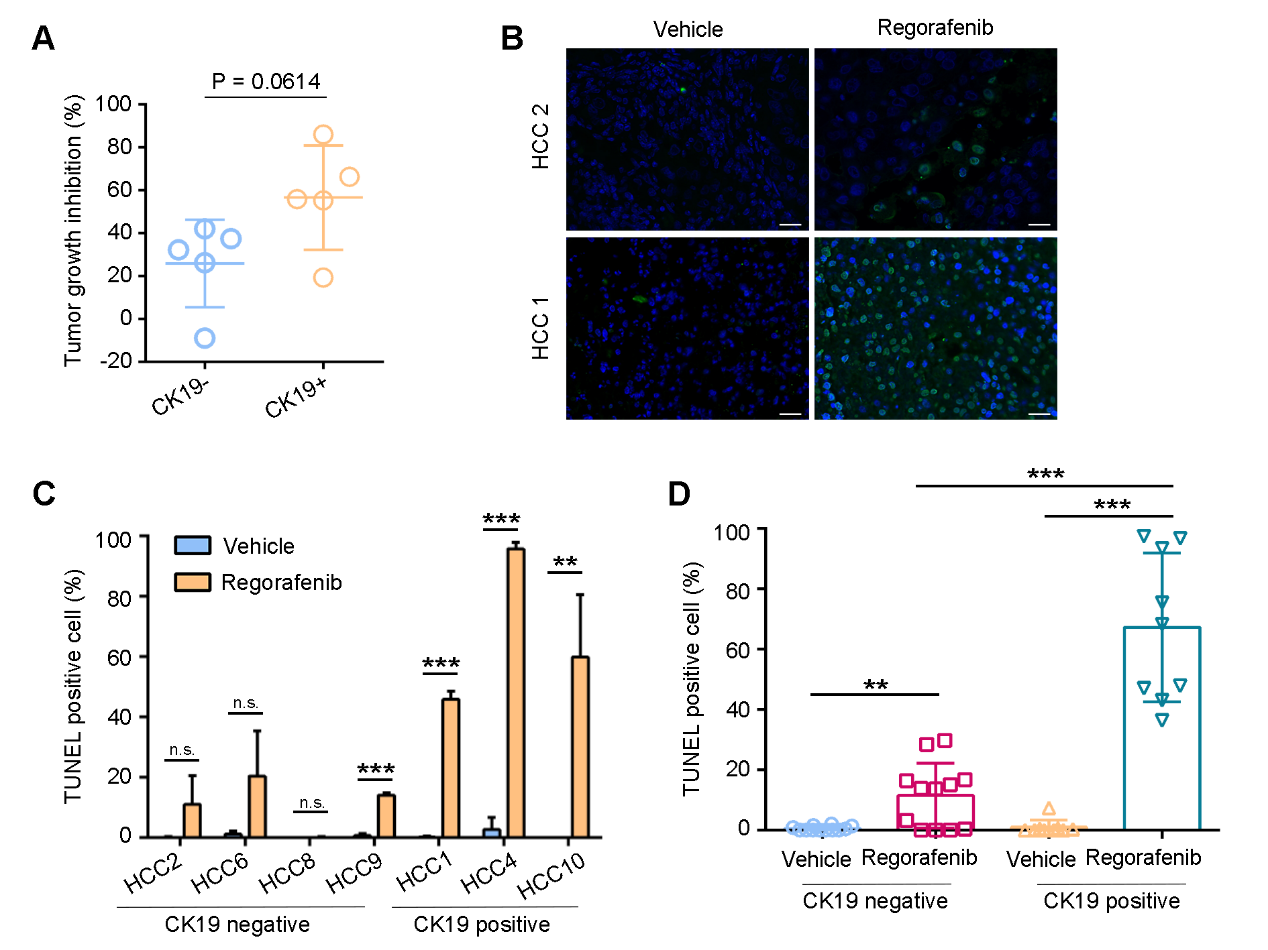


**Supplementary Figure 4.**


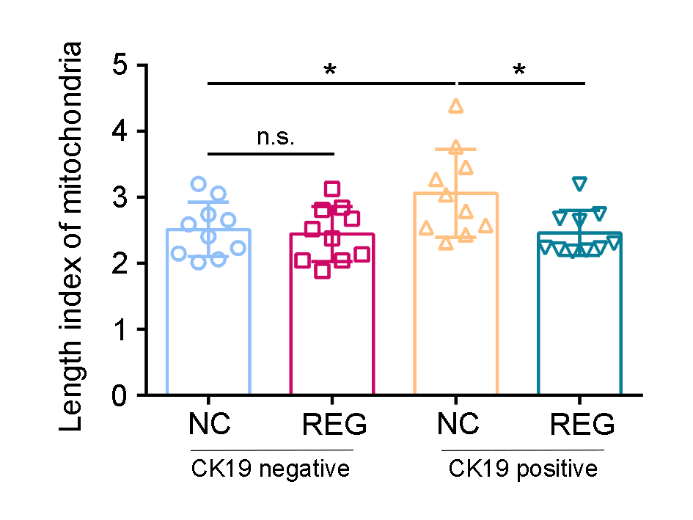


**Supplementary Figure 5.**


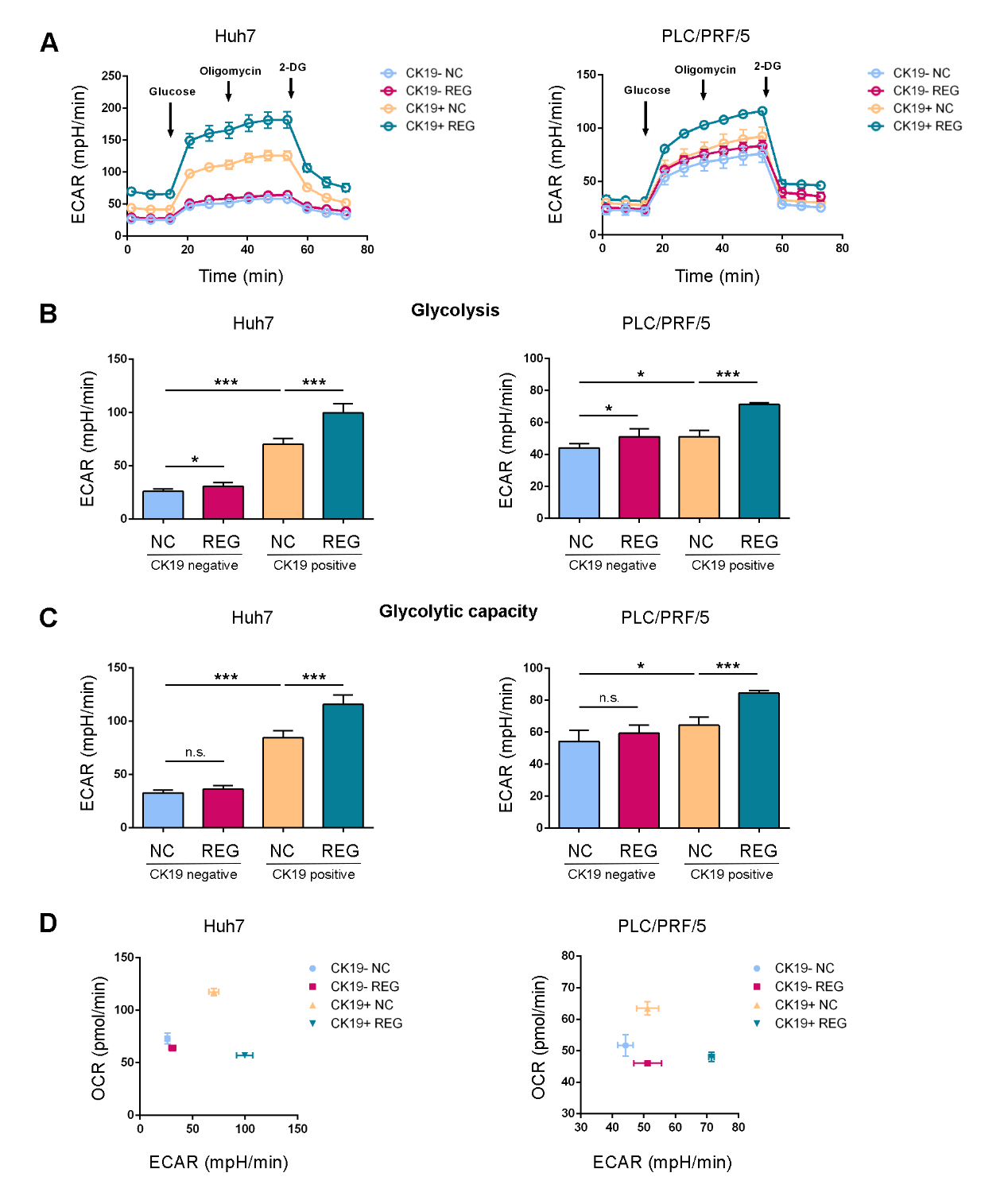


**Supplementary Figure 6.**


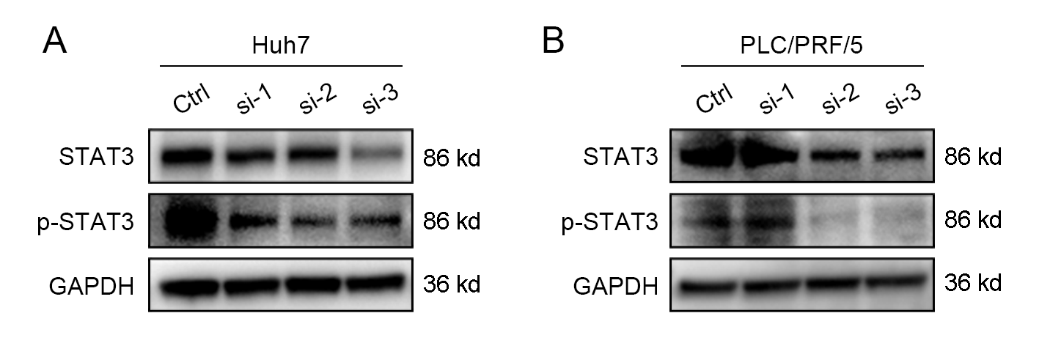

Supplement: Supplementary file 1 — Supplemental material. [file 41419_2021_4320_MOESM1_ESM.docx]
